# Does the Experimenter Presence Affect Verbal Working Memory?

**DOI:** 10.5334/joc.461

**Published:** 2025-09-02

**Authors:** Valérie Camos, Jonathan Jubin, Clément Belletier

**Affiliations:** 1 Université de Fribourg, Département de Psychologie, Fribourg, Switzerland; 2 La Source School of Nursing, HES-SO University of Applied Sciences and Arts Western Switzerland, Lausanne, Switzerland; 3 Université Clermont Auvergne & CNRS, LAPSCO, Clermont-Ferrand, France

**Keywords:** Working Memory, Experimenter Presence, Attentional Maintenance, Concurrent Articulation

## Abstract

Recent studies showed that the presence of the experimenter hinders executive functions. Belletier and Camos (2018) extended these findings to working memory, reporting a detrimental effect of the experimenter presence only when participants performed an aloud concurrent articulation during maintenance. Under such a condition, participants likely relied on an attentional maintenance mechanism rather that an articulatory mechanism, supporting the account of a capture of attention by the social presence. However, other results using the Stroop Task demonstrate an improvement on executive functions (Garcia-Marques & Fernandes, 2024, for a meta-analysis). Thus, the present study aimed at reassessing the impact of experimenter’s presence reported by Belletier and Camos (2018) on a larger sample, with a within-subject manipulation of concurrent articulation, a variation in the secondary task, and the addition of another type of concurrent articulation. In the present study, participants alone or in the presence of the experimenter performed a Brown-Peterson task in which they maintained letters during a 12-second interval, during which they either stayed silent, uttered aloud, or whispered non-sense syllables. They had also to perform either no secondary task, a parity or a location judgement task. Results confirmed Belletier and Camos’ (2018) findings, showing that the experimenter presence hindered memory performance when participants performed a secondary task under any type of concurrent articulation. A silent context or the absence of secondary task preserved recall from the effect of experimenter’s presence.

The presence of others is the elementary brick of the social context, and as such, its effect on performance has been intensively studied (see Belletier et al., 2019; Bond & Titus, 1983, and Guerin, 1993, for reviews). One account suggests that the presence of others automatically captures attention, a cognitive resource needed to fuel executive functions (Baron, 1986; Huguet, et al., 1999), which in turn impacts the performance in the task at hand. This hypothesis is named the distraction conflict hypothesis. Several studies successfully tested this hypothesis by showing that the social presence negatively affects inhibition in the Simon task (Belletier, et al., 2015), cognitive flexibility in a fluency task (Wagstaff, et al., 2008), and voluntary attention control in a visual search task (Wühr & Huestegge, 2010).

Extending the examination of social presence effect on executive functions, Belletier and Camos (2018) tested whether the presence of the experimenter impacts working memory (WM), especially the maintenance of verbal information. Indeed, many WM models suggest that executive attention is needed to maintain information in the short term (see Logie et al., 2021, for a review), and as a consequence recall performance should be impaired by the social presence according to the distraction conflict hypothesis. However, besides the maintenance through an attention-based mechanism, participants could also rely on a non-attentional mechanism of maintenance, the articulatory rehearsal (Baddeley, 1986; Camos, 2015, 2017). Articulatory rehearsal is supported by language processes to maintain verbal information. Hence, if the social presence effect captures attention, WM performance should be depleted under social presence when attentional mechanism is used and not under the use of articulatory rehearsal.

To test this predictions, Belletier and Camos (2018) ran two experiments in which adults maintained series of letters in a Brown-Peterson paradigm in which the 12-second retention interval was either filled by a parity judgment task or unfilled. According to the experiments, during the retention interval, participants either remained silent allowing the use of articulatory rehearsal, or repeated out loud non-sense syllables, a manipulation known to prevent the use of articulatory rehearsal (Norris, et al., 2018; Saito, 1997), and to trigger the maintenance through attentional refreshing (e.g. Camos et al., 2011). While no effects of the social presence appeared when participants remained silent (Experiment 1), participants in the presence of the experimenter recalled less letters than the participants performing the task alone when rehearsal was impaired by a concurrent articulation (Experiment 2). Belletier and Camos’ (2018) results offered first evidence that the presence of the experimenter can hinder WM, as it does for other executive functions. More importantly, these results brought strong support in favor of the distraction conflict hypothesis, because the social impairment effect was not systematically observed, but only in Experiment 2 when participants could not rely on rehearsal and have to maintain memory items through an attention-based mechanism. Indeed, when rehearsal is available, individuals tend to favor its use, even when this mechanism is not optimal (Belletier et al., 2023).

However, another line of research using the Stroop task have systematically established that, on the contrary, the presence of others improves cognitive control in the presence of others (e.g., Huguet et al., 1999; for a meta-analysis see Garcia-Marques & Fernandes, 2024). More specifically, the Stroop interference is reduced when the task is performed in the presence of an observer or co-actor, which can be interpreted as a better capacity to inhibit and to deploy attention (Sharma et al., 2010). Given that inhibition is known, as we have seen, to be strongly linked with WM capacity, the aim of the present study was to reassess the effect of the experimenter’s presence in WM, because Belletier and Camos (2018) is still the only study testing such an effect. These seemingly conflictual results make it all the more necessary to replicate and understand the results on the effects of presence on working memory.

Hence, the present study implemented a similar Brown-Peterson task as the one in Belletier and Camos (2018), but introduced three changes. First, the manipulation of the concurrent articulation was a within-subject design. In Belletier & Camos (2018), this manipulation was between-subjects, the main finding resulting from a between-experiment comparison. The within-subject design allows stronger causal conclusions, and have more statistical power (Maxwell & Delaney, 2004). Second, we also took the opportunity of the present study to vary the type of concurrent articulation by adding a new condition in which the non-sense syllables were whispered. This condition should impair rehearsal and promote the use of the attentional mechanism, without inducing an additional irrelevant speech as can do an aloud articulation. Finally, we varied the concurrent task, half of the participants performed the same parity judgment task on series of digits as in Belletier & Camos (2018), while the other half judged the location of a square on screen (either up or down). Our goal was to assess whether the presence effects on WM are restricted to the parity judgement task or can be extended to another task. The parity judgement task involving digits and most of the participants in Belletier and Camos (2018) being women, the negative stereotype of women in mathematics may have played a role in the effect of the presence of an expert (the experimenter). Moreover, digits are more prone to interfere with letters, which could make memory traces more fragile and context-sensitive (if context is seen as an interference source). Finally, the perceived difficulty of the task could have played a role in the presence effects (Bond, 1982), and we wanted to replicate them with a simpler task. The location judgement task therefore offered the opportunity to neutralize these speculations. In any case, the distraction-conflict theory of Baron hypothesizes that the task requires a significant amount of attention for the emergence of an attentional conflict between the task at hand and social presence. Therefore, the social presence effect should be higher when a secondary task involving attention is added.

Overall, this study also involved a larger sample size. The predictions were the same as in Belletier and Camos (2018). We predicted that, if the experimenter’s presence hinders attention, the reduction of recall performance under the experimenter’s presence should occur when concurrent articulation impairs articulatory rehearsal, but not when doing the task silently. Moreover, the present study allowed us to examine whether the type of concurrent articulation (whispered or aloud) and the type of secondary task (digits or locations) have a different impact on the presence effect.

## Method

### Participants

#### Power analysis

Based on the only presence effect reported in a WM task (Belletier & Camos, 2018, Exp. 2, Cohen’s d = 0.65), a G-power analysis (Faul et al., 2007) with an alpha level of .05, and a desired power of .80, indicated a needed sample size of 78 participants for a t-test with two independent samples.

#### Sample

We hence recruited 24 participants per group, participants being randomly assigned to one of the four groups (2 types of secondary tasks × 2 contexts, i.e., alone vs presence). At the end of testing, analysis was performed on the data of 92 participants,^1^ because four participants were excluded due to poor performance on the secondary task.^2^ They were all undergraduate students from the University of Fribourg (15 males and 81 females; M_age_ = 21.16 years; SD_age_ = 1.95). They received course credit or cinema voucher for their participation. All had normal or corrected to normal vision and were naïve concerning the purpose of the experiment that was presented as a memory study. Ethic approval was obtained from the internal review board, and informed consent was signed by each participant.

### Material and Procedure

**Figure 1:**
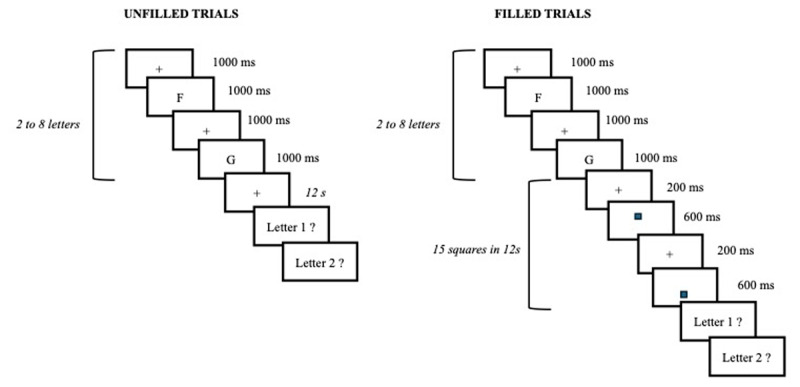
Examples of unfilled and filled trials (here with 2 memory letters). In the filled trials, the secondary task can be a location (here depicted) or a parity (squares were replaced by digits) judgment task. During the 12s interval, participants remained either silent, whispered “ba-bi-bou” or repeat aloud “ba-bi-bou”. The order of these concurrent articulation conditions was counterbalanced across participants.

The task was implemented on a computer using Psychopy (Peirce, 2007). The type of concurrent articulation (silent, whispering and aloud) was manipulated within participants. Each participant performed 70 filled trials and 70 unfilled trials in 3 conditions of concurrent articulation (420 trials in total). The unfilled trials began with to-be-remembered letters that appeared one at the time on the screen for 1 second with no inter-stimuli interval (ISI; Figure 1). The letters were 2 to 8 consonants chosen randomly among consonants (except W, Y, and Z), with 2 trials for each of the 7 lengths. After a beep, participants had to maintain the memoranda during a 12-second blank interval. A second beep indicated the recall of letters, one at the time in their order of presentation by typing them on keyboard. In the filled trials, the blank interval was replaced by a location judgement task on 15 black squares for half of the participants or a parity judgment task on 15 digits for the other half. Digits and squares were sequentially displayed on screen for 600 ms with a 200 ms ISI. Each digit was chosen at random from 1 to 9 and presented at the center of the screen. Each square appeared randomly at the top or bottom of the screen. Participants pressed the left arrow for odd digits, the right arrow for even digits, the up arrow when squares were at the top of the screen and the down arrow if they were at the bottom. Participants were instructed to respond as quickly as possible without sacrificing accuracy. Stimuli were presented at the center of a standard CRT monitor in Lucida Console font with a height of ~1.3° of visual angle, participants being sat at about 60 cm from the screen.

#### Conditions of concurrent articulation

In the silent condition, participants did the task silently. In the whispering condition, participants had to repeat “ba-bi-bou” at the pace of one syllable every 500 ms during the entire retention interval. This concurrent articulation was done in a low voice that was barely audible, began after the presentation of the to-be-remembered letters (at the first beep), and stopped before the recall (at the second beep). The aloud condition was similar, except that the concurrent articulation was made in a high voice.

#### Social presence

Participants were randomly assigned to one of the two contexts. In the alone context, the female experimenter entered in the experimental room at the beginning of each concurrent articulation condition to launch the task and help participants with the instructions. She left the room when the trials started. In the presence context, the experimenter remained in the cubicle and was positioned opposite the participant, in a such a way that she cannot see the participants’ ongoing task, but watched them 60% of the time, as in Belletier and Camos (2018).

#### Training

At the beginning of the experiment, participants were trained on four trials of parity/location (depending on their group) judgment task only (i.e. without memory load) and on two filled trials done silently. Moreover, at the beginning of each concurrent articulation condition, participants were trained on two filled trials. These trials were done silently in the silent condition. On the whispering condition, an audio metronome was first provided as an example of the desired pace, before doing the training trials. It was the same for the aloud condition, except that the articulation was done in a high voice. The experimenter stayed in the experimental room during the training phase to ensure that participants understood the task.

## Results

All analyses were performed with JASP version 0.19.1 (JASP Team, 2024). We used Bayesian statistics to overcome some of the shortcomings associated with null-hypothesis significance testing (Wagenmakers, 2007). Bayesian Analyses of Variance (ANOVAs) were performed using the default settings. The BF_10_ of each model (e.g., main effects only, additive model, and main effects + interaction effects) was obtained by comparing it to the null model. For each dependent variable, we first examined the best model, i.e., the model with the largest BF_10_. Then, we reported the BF_inclusion_ value for each factor in the model (i.e., a main effect or an interaction effect), which indicates the likelihood of the data under models that included a given factor compared to all models stripped of the factor. A BF of 3 or more is considered substantial evidence for the model of interest, a BF below one third is considered substantial evidence for the null model and values around 1 indicate no substantial evidence either way (Dienes, 2014; Jeffreys, 1961). Similarly, we favoured the best model when its probability to account for the data was 3 times greater than the second-best model; otherwise, both models were taken into consideration, and the examination of the BF_inclusion_ of the effects included in the models helped choosing the model to favour.^3^ Finally, it is worth noting that all participants did the filled and unfilled trials, but that half of them had to perform a location judgement task and the other half had to perform a parity judgement task. However, the Bayesian ANOVA method accepts unbalanced designs.

### Secondary judgment task

On average, participants achieved 88% (SD = 8%) correct responses on the secondary judgement task, showing that they complied well with the instructions. An ANOVA with Articulation (silent, whispering vs. aloud) as a within-subjects factor and Context (alone vs. presence) and Task (parity vs. location judgement task) as a between-subjects factors was performed. The three first models did not differ in accounting for the data, BF_10_ between 3.57 × 10^12^ and 1.28 × 10^12^. Examination of the BF_inclusion_ supported only two main effects, favoring then the second-best model, BF_10_ = 1.62 × 10^12^. As expected, performance was better in location (M = 93%, SD = 8) than in parity (M = 84%, SD = 7) judgment task, BF_inclusion_ = 7.56 × 10^7^, and when done silently (M = 90%, SD = 7) than under concurrent articulation (M = 88% and 87%, SD = 9, and 9, in whispering and aloud, respectively), BF_inclusion_ = 2.03 × 10^4^. It should be noted that the BF_inclusion_ for the main effect of Context as well as for the interaction between Articulation and Context were in favor of an absence of effect, BF_inclusion_ = 0.34 and .10, respectively.^4^

### Memory Performance

Memory performance was scored as percentage of correctly recalled letters in their correct serial position as in Belletier and Camos (2018). These percentages were submitted to an ANOVA with Concurrent task (unfilled vs. filled) and Articulation (silent, whispering vs. aloud) as within-subjects factors and Task (parity vs. location judgement task) and Context (alone vs. presence) as a between-subjects factor. Because the two first models accounted similarly for the data, BF_10_ = 2.22 × 10^60^, and 1.46 × 10^60^, we examined BF_inclusion_ of the different effects. Memory performance was better when participants remained silent (M = 83%, SD = 9) than under concurrent articulation (M = 69% and 63%, SD = 12, and 15, in whispering and aloud, respectively), BF_inclusion_ = 2.41 × 10^39^. It was also better in absence of concurrent task (M = 76%, SD = 10) than with a concurrent task (M = 68%, SD = 13), BF_inclusion_ = 1.24 × 10^12^. Additionally, three interactions gathered BF_inclusion_ greater than 3, and they all included Concurrent task, i.e., Concurrent task × Task, BF_inclusion_ = 1.67 × 10^2^, Concurrent task × Articulation, BF_inclusion_ = 6.48 × 10^5^, and Concurrent task × Context, BF_inclusion_ = 3.88. Besides the Concurrent task × Task × Articulation interaction, with a BF_inclusion_ close to 3 (i.e, 2.94), none of the other effects had BF_inclusion_ higher than 3 (from 0.21 to 2.07). Due to the interactions with Concurrent task, we analyzed separately the unfilled and filled trials.

**Figure 2:**
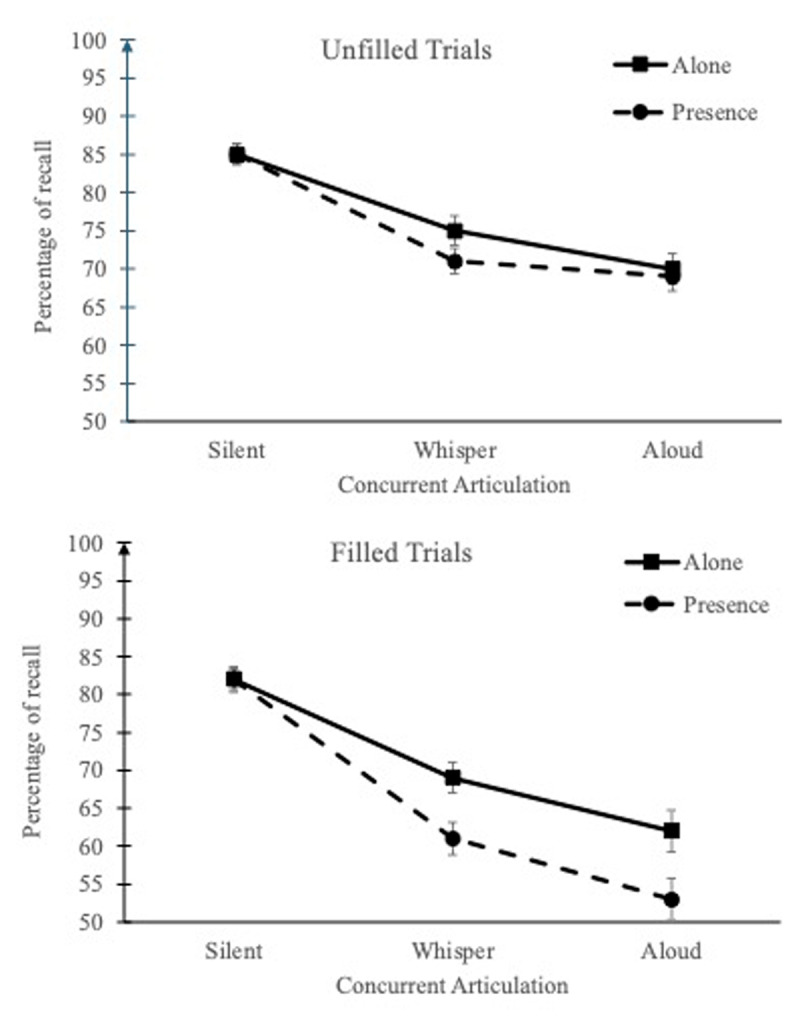
Mean percentage of recall according to Concurrent task (unfilled vs. filled trials), Concurrent articulation (silent, whispered vs. aloud), and Context (alone vs. in the experimenter’s presence). Error bars represent standard errors.

On the unfilled trials, memory performance was better under silent condition (M = 85%, SD = 9) than under concurrent articulation (M = 73% and 69%, SD = 12, and 13, in whispering and aloud, respectively), BF_inclusion_ = 2.19 × 10^28^ (Figure 2), the aloud having a stronger detrimental effect than the whispered, BF_10_ = 6.67. This main effect was the only effect included in the best model, BF_10_ = 1.90 × 10^28^. Although the second-best model added the main effect of Context, BF_10_ = 6.50 × 10^27^, the evidence was in favor of an absence of this effect (M = 76% and 75%, SD = 11, and 9, in alone and presence context, respectively), BF_inclusion_ = 0.34. It could also be noted that BFs_inclusion_ related to the interactions with Context were in favor of the null hypothesis, BFs_inclusion_ between 0.17 and 0.41.

On the filled trials, the pattern of findings was different. Because the two first best models did not differ much, BF_10_ = 8.54 × 10^36^, and 3.31 × 10^36^, we examined the BFs_inclusion_.^5^ As previously reported, a silent condition (M = 82%, SD = 11) benefitted memory performance compared to a concurrent articulation, BF_inclusion_ = 1.96 × 10^34^ (Figure 2), with a stronger detrimental effect in the aloud (M = 57%, SD = 19) than the whispered articulation (M = 65%, SD = 14), BF_10_ = 3.15 × 10^3^. Concerning Context, though the evidence for its main effect was rather weak, BF_inclusion_ = 1.93, evidence in favor of its interaction with Articulation was substantial, BF_inclusion_ = 8.02. Under silent condition, evidence supported an absence of Context effect (M = 82% and 82%, SD = 10, and 12, in alone and presence context, respectively), BF_inclusion_ = 0.22. On the contrary, under concurrent articulation, recall was better in alone context (M = 65%, SD = 15) than in the presence of the experimenter (M = 57%, SD = 15), BF_inclusion_ = 6.53, evidence supporting the absence of interaction with the type of concurrent articulation (whisper vs. aloud, Figure 2), BF_inclusion_ = 0.24.

## Conclusion

Altogether, and despite the introduction of several changes, the current findings are rather similar to Belletier and Camos (2018). The effect of the experimenter on memory performance occurred when an active maintenance is called by the distraction of a secondary task and only under a concurrent articulation. This latter should have impaired articulatory rehearsal and verbal memoranda should have been attentionally maintained. It could be additionally noted that the effect experimenter’s presence was similar under the different concurrent articulations, either whispered or aloud, although the aloud articulation had a stronger detrimental effect on recall performance. These findings provide then additional support to the distraction conflict hypothesis. Moreover, presence effects did not depend on the nature of the secondary task, but only on whether it was present or not. This also seems to suggest that any attention-capturing secondary task is likely to give rise to a presence effect. It should be noted that in the study by Belletier and Camos (2018), we had observed an effect of presence even on unfilled trials (without a secondary task), which had led us to assume a strictly additive effect of social presence. This is not the case in the present study, in which presence effects are only observed in filled trials (with secondary task). However, this finding echoes a proposal by Baron (1986), who notes that the task must capture a non-negligible amount of attention for an attentional conflict between the task at hand and social presence to emerge. Nevertheless, future studies could explore this issue in more detail by manipulating the attentional demand of the task more finely.

Interestingly, we found a very different pattern in children aged from 5 to 11 years, in a recent study implementing a similar WM task (Camos et al., 2025). The rationale of this study was that WM recall performance of children who are able to use attentional refreshing should also be impaired by the presence of the experimenter, and not younger children. Contrary to these expectations, recall was not impacted by the experimenter’s presence in children of all age groups. This finding could result from developmental differences between adults and children in the impact of the experimenter presence. Before examining what can drive such developmental differences, the present study allowed us to reassess the effect of the experimenter’s presence in adults’ WM, as Belletier and Camos (2018) was still the only study testing such an effect. By confirming our previous results in adults, the present study calls for further examination on the developmental path of the presence effect as several hypotheses could be tested (see Frick, 2025, for a review).

An alternative explanation to the distraction-conflict hypothesis could be that the experimenter’s presence may influence how diligently participants perform the concurrent articulation task, rather than affecting attention or rehearsal processes. When alone, participants might not consistently repeat the syllables, while the presence of an experimenter could increase compliance due to being observed. This would still be an experimenter effect, but it would enhance performance rather than impair it, contrary to the distraction-conflict hypothesis. To rule out this possibility, future studies could use objective measures of articulation performance. Finally, our results call in favor of a better report of the experimenter’s behavior in WM studies, as the experimenter presence might influence some trials but not others, in the same participants.

## Data Accessibility Statement

Data are available on OSF: https://osf.io/wy5fg/?view_only=65fb7088d123490b9dd40fb7ef3bd11b.

## Footnotes

Twenty-four participants in alone-parity group, 22 in presence-parity, 22 in alone-location and 24 in presence-location group.

Two were excluded in the presence context, because they had less than 65% correct responses in the parity judgement task, and two in the alone context, because they had no correct responses in the location judgement task (probably due to misuse of keys to respond).

To avoid switches between different types of BFs, we only reported BF_10_ and BF_inclusion_. Please note that BF_01_ and BF_exclusion_ can be computed with the reported BF values: BF_01_ = 1/BF_10_ and BF_exclusion_ = 1/BF_inclusion_.

The analysis of response times (RTs) was not included here, because, due to errors in data collection, the available RTs were not cleaned up. Nevertheless, they are available on the OSF page.

Besides the effects discussed in the main text, there was evidence in favor of the main effect of Task with better recall in a location (M = 64%, SD = 13) than a parity (M = 72%, SD = 11) judgment task, BF_inclusion_ = 8.89, and its interaction with Articulation, showing a stronger effect of Task under an aloud articulation, BF_inclusion_ = 10.99.
